# Cerebral vasculitis associated with an Echovirus 6 meningoencephalitis—Case report and review of the literature

**DOI:** 10.1002/ccr3.1963

**Published:** 2018-12-18

**Authors:** Laura Cauwenberghs, Peggy Bruynseels, Nathan Demeyere, Machiel van den Akker

**Affiliations:** ^1^ Department of Pediatrics ZNA Queen Paola Children’s Hospital Antwerp Belgium; ^2^ Department of Pediatrics Antwerp University Hospital Antwerp Belgium; ^3^ Department of Microbiology ZNA Middelheim Antwerp Belgium; ^4^ Department of Radiology ZNA Middelheim Antwerp Belgium; ^5^ Department of Pediatric of Hematology Oncology UZ Brussel Brussels Belgium

**Keywords:** cerebral vasculitis, children, Echovirus 6, enterovirus encephalitis, pediatric stroke

## Abstract

When a previously healthy child presents to the hospital with a stroke, generally a Varicella zoster virus vasculopathy seems most likely. However, other causes of a local cerebral vasculitis are possible and need to be explored.

## 1 INTRODUCTION

Pediatric stroke is often attributed to Varicella vasculitis. However, other causes of a local cerebral vasculitis are possible and need to be explored. We present an infant with a hemiparesis and an ischemic lesion on MRI due to a local Echovirus 6 vasculitis.

Although enterovirus meningitis is common in children, it has rarely been reported to cause focal neurologic abnormalities such as seizures and hemiparesis. The pathogenesis for this presentation remains unclear; however, focal inflammation, vasculitis, and infarction have been described.^1^ Bacterial meningitis, Herpes simplex encephalitis, and Varicella zoster virus vasculopathy need to be excluded in children who present with focal neurologic signs and fever. We report a case of an 8‐month‐old girl with an unusual neurologic complication of an Echovirus 6 infection.

## 2 CASE PRESENTATION

An 8‐month‐old female presented to the emergency department of our hospital with a right‐sided hemiparesis and a mild right‐sided facial paresis, which had been progressive since one day. Further clinical examination was normal and there were no apparent skin lesions. The week before, she had experienced high fever for two days followed by irritability, anorexia, and low‐grade fever. She was born full term via uncomplicated vaginal delivery after a normal pregnancy and was the third child of healthy non‐consanguineous parents from African European descent. Besides an uncomplicated Varicella infection at the age of 6 months, anamnesis and family history did not reveal any relevant information.

**Figure 1:**
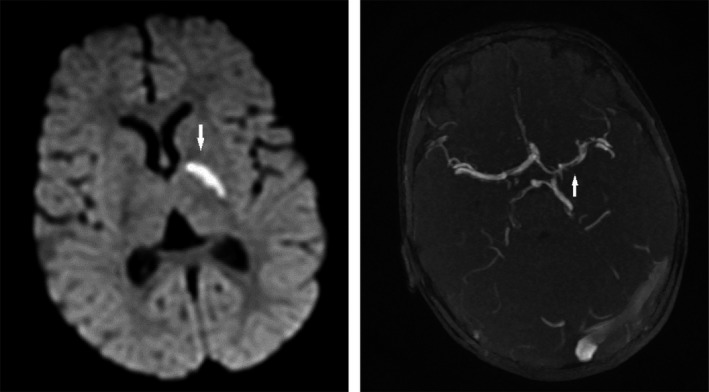
MRI brain: on the left, ischemic injury in the left capsulo‐thalamic region (T2‐weighted image, axial plane), while on the right, the angio‐MR image showing a thinner and irregular aspect of the left arteria cerebri media

Laboratory investigation, including complete blood count, C‐reactive protein, liver function tests, kidney function, and electrolytes, was within the normal range. Computed tomography of the brain did not show any abnormalities, whereas the magnetic resonance imaging (MRI) with angiography of the brain revealed a (sub) acute ischemic lesion of the left capsule‐thalamic region with irregularities of the left arteria cerebri media, suggestive of vasculitis (Figure 1). The vasculitis lesion can be classified as benign (single, concentric, graduated, and smooth aspect of the lesion) and proximal (location on the M1 segment of the left middle cerebral artery). Electroencephalography was normal. Lumbar puncture was done showing normal liquor opening pressure. Examination of liquor indicated an elevated white blood cell count (186 cells/mm^3^) with normal glucose (55 mg/dL) and protein levels (20 mg/dL). While in‐house PCR for Varicella zoster virus and Herpes simplex virus were negative, PCR for enterovirus (GeneXpert, Cepheid) was positive. Bacterial culture remained negative. The sample was sent to the national reference center, and the strain was typed as Echovirus 6 by sequencing. Echocardiography and Doppler ultrasound of the lower limbs and abdomen were normal. Hereditary and acquired hypercoagulability workup (activated partial thromboplastin time, prothrombin time, fibrinogen, D‐dimers, antithrombin III, protein C activity, activated protein C resistance, protein S activity, prothrombin G20210A mutation) was normal. Lupus anticoagulant was negative. Since the focal origin of the vasculitis, and the suspected cause of this, a brain biopsy was not considered.

Intravenous methylprednisolone (1 mg/kg/d, 5 days) and acyclovir (30 mg/kg/d, 14 days) were administered as initial therapy. Even though PCR for Varicella zoster and Herpes simplex were found to be negative, the treatment with corticosteroids and acyclovir was completed because of the clear neurologic deficit and the history of the Varicella zoster infection. Because of the severity of the neurologic deficit and in anticipation of the results of the coagulopathy screening, subcutaneous enoxaparin (2 mg/d) was started. Neurologic abnormalities recovered slowly during the following weeks. Enoxaparin was discontinued after eight weeks, and oral aspirin (30 mg/d, for two years) was initiated.

## 3 DISCUSSION

Enterovirus infection usually presents as a mild febrile illness, but is also known to cause meningitis in children. Rarely, it presents with acute focal neurological symptoms such as seizures, hemiplegia, and focal weakness. In previous literature, this clinical image has been referred to as focal encephalitis. Cerebral vasculitis has been proposed as an underlying cause for cerebral infarction in this particular disease.^2^, ^3^, ^4^ In 2016, Benschop et al,^5^ report an increase in Echovirus 6 infections associated with general neurological symptoms such as aseptic meningitis, encephalitis, and convulsion.

A PubMed search was conducted in the English‐language literature using the keywords “focal neurological signs” and “enterovirus infection” in the age group 0‐16 years between 1975 and 2016. Table 1 gives a summary of the collected cases, including our own case.^2^, ^3^, ^4^, ^6^, ^7^, ^8^, ^9^, ^10^ In all but one case, the symptoms improved spontaneously.

**Table 1:** Reported cases in the English‐language literature of children (0‐16 y) between 1975 and 2016 with focal neurological signs and an enterovirus infection

Reference	Demographic descriptive	CSF—WBC/mm^3^; enterovirus	Serology—enterovirus	Neurologic manifestations	Imaging	Outcome
Current case	F; 8 mo	186; Echovirus 6 (PCR)	‐	Acute right‐sided hemiplegia	MRI focal changes with vasculitis	Complete resolution at 2 mo
Shiohama et al (2015)^10^	M; 9 mo	234; Coxsackie B5 (PCR and virus isolation)	‐	Clustered seizures	MRI normal	Complete resolution
Tsai et al (2004)^3^	M; 2 mo	UK	Enterovirus 71	Focal seizures and right hemiparesis	MRI focal changes with vasculitis	UK
Ayala‐Curiel et al (2003)^6^	F; 20 mo	80	Coxsackie B	Acute left‐sided hemiplegia	CT scan normal	Complete resolution
Wakamoto et al (2000)^4^	M; 4 y	46	Coxsackie A3	Seizures, aphasia with left‐sided facial weakness	SPECT changes (CT/MRI normal)	Complete resolution at 1 mo
Modlin et al (1991)^8^	M; 12 y	24; Coxsackie A5 (EIA)	Coxsackie A5	Focal seizures and left‐arm weakness	CT scan focal changes	Complete resolution
	M; 13 y	63	Coxsackie A5	Jacksonian seizure	CT scan normal	Complete resolution
	M; 4 y	102	Coxsackie A5	Focal seizures	CT scan normal	Complete resolution
	F; 7 wk	700	Coxsackie B2	Focal seizures	CT scan normal	Complete resolution at 18 mo
Peters et al (1979)^9^	M; 5 y	Echo virus (IIFT)	Echo virus 25	Hemichorea	CT scan focal changes	Near complete resolution
Chalhub et al (1977)^7^	F; 3 mo	Elevated; Coxsackie A9 (virus isolation)	‐	Focal seizures and hemiplegia	99Tc scan and CT scan changes	Porencephaly, seizures, mental retardation, hemianopsia
Roden et al (1975)^2^	F; 16 mo	21; Coxsackie A9 (virus isolation)	‐	Acute hemiplegia	99Tc scan focal changes	Residual hemiparesis at 1 mo

CT, computerized tomography; EIA, enzyme immunoassay; F, female; IIFT, indirect immunofluorescent technique; M, male; MRI, magnetic resonance imaging; SPECT, single‐photon emission computed tomography; UK, unknown.

In our patient, brain MRI with angiography showed an ischemic lesion on the left side with associated vasculitis of the left arteria cerebri media. This was also reported by Tsai et al,^3^ describing a 2‐month‐old boy with focal seizure and right hemiparesis. MRI and angiography showed vasculitis in the left anterior cerebral artery with cerebral infarction. To our knowledge, the presence of cerebral vasculitis in enterovirus infection has not been reported in previous cases.

Two other case reports, however, have also proposed local cerebral vasculitis as a cause of vascular occlusion. Wakamoto et al report a 4‐year‐old male with focal Coxsackie A3 encephalitis who presented with seizures and acquired aphasia. Brain single‐photon emission computed tomography (SPECT) disclosed hypoperfusion in the right frontal lobe, which completely resolved on follow‐up imaging.^4^ They propose that these mild ischemic changes are most probably caused by local cerebral vasculitis. Roden et al describe a case of a 16‐month‐old girl with an acute hemiplegia associated with Coxsackie A9 encephalitis. Technetium‐99 brain scans revealed a lesion within the region of the right middle cerebral artery.^2^ They also propose that the most likely underlying mechanism is a vascular occlusion caused by focal vasculitis. Both case reports were not able to identify the presence of vasculitis on imaging.

Until now, the underlying pathogenesis of focal enterovirus encephalitis remains under speculation. Older cases described, suggest direct cytotoxicity of the virus causing focal necrotizing encephalitis.^7^, ^9^

In general, focal neurological symptoms and focal cortical hyperintensity on brain MRI are associated with a poor neurological outcome.^11^ Although the extent of the ischemic lesion seen on MRI in our patient was substantial, the general course of the disease remained benign. Only one of the patients described with focal neurological symptoms in enterovirus meningoencephalitis had persistent neurological defects.^7^ As in our patient, all other patients made a complete or near complete recovery. It is important to differentiate this self‐limiting disease from intracerebral Varicella zoster virus vasculopathy, in which intravenous methylprednisolone and acyclovir are often added to the therapy.

## 4 CONCLUSION

When a previously healthy child presents to the hospital with a stroke, generally a Varicella zoster virus vasculopathy seems most likely. However, other causes of a local cerebral vasculitis are possible and need to be explored. We present an infant with a hemiparesis and an ischemic lesion on MRI due to a local Echovirus 6 vasculitis. The clinical evolution was favorable.

## CONFLICT OF INTEREST

The authors have no conflicts of interest to disclose.

## AUTHOR CONTRIBUTIONS

LC: drafted the initial manuscript and approved the final manuscript as submitted. PB: reviewed the manuscript critically and approved the final manuscript as submitted. ND: participated in the writing of the manuscript, critically reviewed the manuscript, and approved the final manuscript as submitted. MA: carried out the initial analyses, coordinated and supervised the writing of the manuscript, critically reviewed the manuscript, and approved the final manuscript as submitted.
